# Exploring Nadolol as a Therapeutic Option for Infantile Hemangioma: A Narrative Review

**DOI:** 10.7759/cureus.94995

**Published:** 2025-10-20

**Authors:** George Chamoun, Jonah P Gutierrez, Rafael Sad do Valle, Kayla Fure, Alyssa Forsyth, Aqsa Kanwal, Hunjung Choi

**Affiliations:** 1 Dermatology, Texas College of Osteopathic Medicine, Fort Worth, USA; 2 Pediatrics, Texas College of Osteopathic Medicine, Fort Worth, USA; 3 Dermatology, Michigan State University College of Osteopathic Medicine, Detroit, USA; 4 Dermatology, Columbia School of Medicine, Columbia, USA

**Keywords:** infantile hemangioma, nadolol, nadolol therapy, pediatric vascular tumor, propranolol therapy, treatment efficacy and safety

## Abstract

Infantile hemangioma (IH) is a common vascular tumor of infancy, characterized by rapid early growth and gradual spontaneous involution. Propranolol remains first-line therapy for complicated IH but is limited by adverse effects and contraindications in some infants. Nadolol, a nonselective beta-adrenergic antagonist with a longer half-life and reduced CNS penetration, has been proposed as an alternative. Evidence suggests these pharmacokinetic properties may improve safety, reduce neurocognitive side effects, and enhance adherence while achieving comparable efficacy to propranolol. However, data are limited, particularly regarding long-term safety and standardized dosing protocols. Further controlled studies are needed to define nadolol’s role in IH management.

## Introduction and background

Infantile hemangioma (IH) is a common vascular tumor of infancy that typically does not appear at birth but develops within the first few months of life. These lesions may present as flat or raised growths on the skin with a red-blue hue. Often referred to as a “strawberry mark,” IHs undergo a rapid proliferative phase followed by a gradual involution phase [1]. While most IHs are benign and resolve without intervention, their location may sometimes pose significant challenges. Although they are primarily located on the skin surface, they can also involve internal organs, such as the airway, leading to breathing difficulties, or periocular regions, potentially causing blindness and other vision problems [1]. Early recognition and appropriate management are essential to prevent functional impairment and long-term complications.

Propranolol, a nonselective beta-adrenergic antagonist, is the first-line treatment for IH due to its established efficacy in reducing tumor size and achieving favorable clinical outcomes. However, propranolol is associated with adverse effects, including hypotension, bradycardia, decreased appetite, sleep disturbances, hypoglycemia, shortness of breath, irritability, and fatigue, particularly in infants [2]. These potential side effects, along with known contraindications such as reactive airway disease or cardiovascular conditions, can further limit its use [3]. In these cases, where propranolol is contraindicated, poorly tolerated, or ineffective, exploring alternative beta-blockers with a distinct pharmacological profile, such as reduced lipophilicity, becomes imperative [4]. Emerging research suggests that nadolol, another nonselective beta-adrenergic antagonist, may offer a safer and more effective treatment option for these patients.

Nadolol exhibits pharmacokinetic properties that offer advantages over the conventional use of propranolol. For instance, its longer half-life and reduced penetration into the CNS minimize the risk of neuropsychiatric side effects, such as irritability and sleep disturbances [5]. Furthermore, its extended half-life decreases the need for frequent dosing (given once daily), improving adherence and making it easier for caregivers to manage [5]. Additionally, its pharmacokinetic profile supports more favorable therapeutic outcomes, including the absence of known sympathomimetic properties, minimal myocardial depressant activity, and a half-life of 12-24 hours [4]. Since nadolol is minimally metabolized by the liver and is excreted unchanged by the kidneys, it is preferable in infants with hepatic impairment, where propranolol metabolism may be compromised [5]. These characteristics make nadolol a compelling alternative in clinical scenarios where safety, tolerability, and ease of administration are paramount.

Indications for using nadolol in the treatment of IH include cases where hemangiomas pose a risk of causing serious damage to a part of the body (eye, liver, and airway), are ulcerated, or have the potential to cause permanent scarring [6]. Preliminary studies have reported comparable efficacy of nadolol in reducing hemangioma size while demonstrating a favorable safety profile. However, comprehensive investigations into the long-term safety, optimal dosing regimens, and appropriate patient selection criteria remain limited, necessitating further research.

This review aims to critically evaluate the current evidence regarding the efficacy and safety of nadolol as a therapeutic option for the treatment of IH. A targeted literature search was conducted using databases and search engines such as PubMed and Google Scholar to identify peer-reviewed studies, reviews, and case reports relevant to nadolol use in IH. Specifically, it provides information regarding the pharmacokinetic properties of nadolol, compares its therapeutic outcomes with propranolol, assesses safety and tolerability for use in infants, and identifies gaps in the literature to guide further research. By synthesizing available data, this review offers insights into the potential role of nadolol as an alternative treatment for IH, supporting clinical decision-making and optimizing patient care.

## Review

IHs

Pathophysiology and Clinical Presentation

With prevalence rates estimated at up to 5%, IHs are the most common benign vascular tumors of infancy. These lesions are characterized by a pattern of rapid growth followed by spontaneous involution, with most cases not requiring medical therapy [7]. Unlike congenital hemangiomas, which are present at birth, IHs typically arise later in infancy, generally around the first four weeks of life. However, they may be preceded by various types of precursor lesions. Generally, IHs follow a natural course characterized by three phases: the early proliferative phase, marked by rapid growth; the plateau phase, during which lesions maintain their size; and the involution phase, lasting from several months to potentially several years. Despite extensive research, the exact etiology of IHs remains poorly understood. It is currently believed that hypoxic conditions lead to increased expression of GLUT1 and VEGF, resulting in the mobilization of endothelial progenitor cells [8]. A study by Yu et al. (2004) revealed the presence of CD133, a progenitor cell marker, as well as KDR, a marker for endothelial cells, in proliferating hemangiomas, suggesting a role for endothelial progenitor cells in the expansion and proliferation of IHs [8]. In 2025, the International Society for the Study of Vascular Anomalies divided IHs into four groups according to features such as size, location, and treatment: localized, segmental, indeterminate, and multifocal [9]. Notably, individuals with segmental IH are more likely to require treatment due to an increased likelihood of complications [10,11]. Given the diverse array of presentations and complications associated with IH and its relatively high prevalence, further exploration of its complications is essential.

Impact of IH on Health and Quality of Life

The most common complications associated with IH include ulceration, disfigurement, obstruction, and functional impairment, with ulceration being the most frequent, occurring in about 25% of cases [12]. Ulcerations may involve skin or mucosal surfaces, causing significant pain, bleeding, risk of infection, and almost always resulting in permanent scarring, particularly when involving the scalp, neck, perioral, perineal, perianal, and intertriginous sites. IHs also pose aesthetic concerns due to disfigurement, especially when located in prominent areas such as the face, parotid region, and chest in girls. Functional impairments can include visual disturbances such as ptosis, strabismus, anisometropia, or astigmatism due to periocular involvement, as well as interference with feeding due to lip or mouth involvement [7]. Importantly, life-threatening complications may occur, including respiratory distress and swallowing difficulties from airway involvement (subglottic lesions), hepatic hemangiomas (usually asymptomatic but potentially associated with macrovascular shunting leading to high-output cardiac failure and hypothyroidism), and significant hemorrhage [6,13]. It is believed that subtype, size, location, and, most significantly, morphology are important predictors of complications and prognosis [14]. Although most IH lesions are benign and self-limiting, the potential for severe complications, including life-threatening emergencies, underscores the necessity of a thorough evaluation of each case, considering lesion location, size, and involvement of surrounding anatomical structures.

Current management strategies

Mechanisms of Propranolol

Propranolol is considered the first-line treatment for IHs requiring systemic therapy and is currently the only FDA-approved treatment option. Propranolol is a lipophilic, nonselective β1 and β2 receptor antagonist that is capable of crossing the blood-brain barrier and is commonly available to treat IH as an oral hydrochloride solution known as Hemangeol. The efficacy of propranolol in treating IHs was discovered incidentally while treating neonates with cardiomyopathy [15]. This subsequent discovery revolutionized the medical management of IHs. Propranolol has been deemed to be highly efficacious in reducing the size and progression of IH, irrespective of where the lesion is located. While it has been proposed that propranolol exerts the greatest benefit during the proliferative phase, when hemangiomas grow most rapidly, clinical trial data demonstrate meaningful efficacy even after this phase. In the pivotal New England Journal of Medicine phase 2-3 study of 460 infants, 60% of those treated with propranolol achieved complete or nearly complete resolution at 24 weeks compared with only 4% in the placebo group (P < 0.001). Additionally, 88% showed improvement as early as week 5, with durable responses sustained through 96 weeks of follow-up, confirming its effectiveness well beyond the proliferative period [16].

The exact mechanism of action of propranolol in the treatment of IH remains poorly understood, with various proposed mechanisms. In one study, propranolol inhibited the SOX18 transcription factor, which is important in vascular development and tumor angiogenesis. Another proposed mechanism consists of its therapeutic action being based on lowering cAMP and activating the MAPK pathway downstream of β1 and β2 receptors in hematopoietic stem cells [17]. Irrespective of the mechanism, treatment with propranolol ultimately results in the inhibition of cell proliferation and the upregulation of apoptosis in a dose-dependent fashion [18]. An additional study found that propranolol increased apoptotic markers such as Bax and Caspase3 and decreased Bcl-2 expression in endothelial IH cells [19]. Various additional mechanisms have been proposed; however, no specific mechanism of action is currently identified as the core mechanism by which propranolol exerts its effects on IH cells.

Adverse Effects of Propranolol

While treatment with propranolol significantly diminished tumor growth and size, the adverse effects must be taken into consideration when utilizing this medication. Many patients on propranolol have documented hypotension, bradycardia, hypoglycemia and seizures, bronchospasm, sleep disturbance, peripheral coldness, and gastrointestinal disturbance. The most common adverse effects reported in clinical trials were peripheral coldness, diarrhea, and sleep disturbances. Additionally, decreased heart rate and low blood pressure were also commonly reported concerning treatment with propranolol. Severe adverse effects were rare in clinical trials but often resulted in discontinuation of therapy. Two cases of atrioventricular block were reported, one of which progressed to fatal heart failure. Bradycardia was observed in two trial participants; however, additional cases have been documented in the literature. In most instances, bradycardia was considered mild, though some required inpatient observation or dose titration. Hypotension was generally mild, except for a few life-threatening cases, which resolved after discontinuation or dose reduction [20]. While severe adverse effects of propranolol use are rare, there have been several published deaths of children occurring during propranolol treatment. Most of these have been sudden, unexplained deaths not directly related to other medical causes. Of these cases, toxicology reports showed levels of propranolol within the therapeutic range. Postmortem glucose levels were not obtained, as they have no prognostic value in premortem glucose. Some of these infants also had risk factors for sudden infant death syndrome, which are not uncommon in the general population [21]. In summary, several potentially corresponding factors lead to uncertainty regarding the actual correlation between propranolol use and sudden unexplained death.

Alternative Therapies

Corticosteroids can be used in the treatment of IH; however, they are not FDA-approved for IH treatment and are less effective than propranolol in monotherapy. The mechanism by which corticosteroids slow the growth of IH is by decreasing VEGF-A expression, which is present during the proliferative phase of IH growth. Monotherapy with corticosteroids is rarely used, as it is less efficacious than propranolol, often with greater adverse effects associated with exogenous steroid use. Combination therapy of corticosteroids with propranolol has been shown to be efficacious in reducing proliferation and size of IH, potentially indicated for IH that occur in locations that can be life- or function-threatening, such as IH obstructing the airway or vision [17]. Pulsed dye laser (PDL) can be used as a treatment option for IH, working to target blood vessels and induce thermal damage to the blood vessels within the tumor. Damage to surrounding tissue can be minimized with the use of the flash lamp-pumped PDL to emit light that is mainly absorbed by hemoglobin within superficial vessels. In a systematic review, PDL demonstrates clinical efficacy in treating IH with minimal adverse effects. Subcutaneous and deep hemangiomas have not shown significant benefit from this treatment, as the light cannot penetrate deeper tumors effectively [22]. The use of laser therapy is not considered first-line therapy, but its use can be beneficial in treating tumors unresponsive to other therapies.

For hemangiomas that have a suboptimal clinical response to pharmacologic treatment, surgical resection may be a safe alternative. Adverse events related to the surgical excision of hemangiomas are reported to be low, without serious complications being reported. Timing of surgery seems to have the highest indication for overall risk of complications, with children at or under the age of three having the greatest risk for adverse effects [23]. The anatomic location of the hemangioma plays a role in the satisfaction of surgery, with those located on the head and neck more likely to be associated with undesirable outcomes, potentially suggesting earlier surgical intervention. Overall, surgical intervention for hemangiomas is typically reserved for more severe hemangiomas or those that are located on the head, neck, or in life-threatening locations.

Nadolol overview

Nadolol is a synthetic nonselective β-adrenergic receptor blocker with inverse agonist activity. It competitively inhibits β-1 receptors located in the heart and vascular smooth muscles, blunting the effect of catecholamines on these targets [5]. This action results in decreased heart rate, reduced myocardial contractility, and lower systemic vascular resistance. Nadolol also antagonizes β-2 receptors in bronchial and vascular smooth muscle, though its reduced penetration into the CNS distinguishes it from some other nonselective beta-blockers. These pharmacologic characteristics have generated interest in its potential role for treating IHs in select patient populations.

Based on a study completed in 2021 comparing the effectiveness of treatment for subglottic hemangiomas between propranolol and nadolol, the study found that propranolol had increased solubility into the CNS, cardiac effects such as hypotension and bradycardia, and additional side effects such as bronchospasm and hypoglycemia. Given these side effects, nadolol was found to be a promising alternative due to its lower lipophilicity, which limits penetration into the CNS and thereby reduces neurologic side effects. Also, nadolol demonstrated fewer cardiotoxic effects and has a longer half-life, which can be advantageous in infants with IHs by allowing for less frequent dosing and potentially improving compliance. An elevated half-life can be beneficial in infants who get IHs because it can reduce medication dosing and thus increase compliance. The study additionally found no major differences in the side effect profile, with one patient treated with propranolol experiencing vomiting and two patients treated with nadolol having mild hypotension, but this difference across groups was not statistically significant [24]. These findings suggest that nadolol could be considered a viable first-line alternative to propranolol for subglottic hemangiomas, particularly in patients at higher risk for CNS or cardiac side effects. Despite the results of this study showing some favorability towards nadolol as the choice for treatment of subglottic hemangiomas, the study is limited due to its small sample size and retrospective nature.

Nadolol’s duration of action of an oral tablet is approximately 20-24 hours, with an approximate 30% absorption. Nadolol's volume of distribution is between 147 and 157 L, increasing the oral dosage requirements of the drug, with formulations available in 20 mg, 40 mg, and 80 mg. The drug is approximately 30% protein-bound [25]. Nadolol’s metabolism after the first pass is still unclear. Still, excretion is known to occur through the kidneys, with nadolol primarily being excreted in the urine with about 60% elimination and then about 15% in the feces. This is important in infants with renal impairment, because in that instance, nadolol would be contraindicated to avoid accumulation and toxicity.

The greatest differences between nadolol and propranolol are the inherently longer duration of action of nadolol, which allows once-daily dosing, its excretion through the kidney, and its inability to cross the blood-brain barrier, which prevents any sleep disturbance or irritability.

Nadolol for IH: a critical review of efficacy, safety, and comparative effectiveness

As research expands in the treatment of IHs, nadolol has emerged as a credible therapeutic alternative to propranolol for select patient populations. While much of the foundational knowledge around beta-blocker therapy is anchored in propranolol’s proven efficacy, growing evidence suggests that nadolol may be similarly effective and, in specific contexts, better tolerated. This section reviews recent clinical evidence, comparative studies, safety data, and real-world observations relevant to using nadolol in IH management.

Efficacy in Clinical Context

Nadolol’s efficacy in treating IH has been demonstrated in both controlled trials and smaller observational studies. In a landmark randomized clinical trial, Pope et al. found that nadolol was non-inferior to propranolol over 24 weeks of treatment, showing similar improvements in lesion size and color [4]. Specifically, at 24 weeks, the nadolol group exhibited a mean reduction of 8.8 mm (95% CI: 2.7-14.9) in lesion size and 17.1 mm (95% CI: 7.2-30.0) in color improvement compared to baseline. These differences were maintained at 52 weeks, with nadolol continuing to show superior rates of involution. The coefficient of involution per week was 2.4 times higher with nadolol than with propranolol, indicating a faster and more pronounced response [4].

Clinical data from retrospective and prospective studies support nadolol’s efficacy in managing problematic IHs. A well-conducted single-institution retrospective cohort study by Randhawa et al. evaluated 44 infants treated with oral nadolol for IHs associated with functional impairment or cosmetic disfigurement [26]. The study demonstrated a mean improvement of 91.8% ± 11.1% based on blinded assessment using a Visual Analogue Scale, with 95% of patients achieving at least 50% lesion involution. Notably, 89% reached ≥75% improvement, with median times of 2.9 months and 3.7 months, respectively, to reach these milestones. Importantly, younger age at treatment initiation was positively correlated with response magnitude (p < 0.05). These findings align with the broader consensus that early intervention during the proliferative phase of IH yields the most favorable outcomes and suggest that nadolol may be particularly effective when initiated promptly.

Comparative Effectiveness and Safety

While both nadolol and propranolol are nonselective beta-adrenergic antagonists, their pharmacokinetic profiles diverge in clinically meaningful ways. Nadolol’s extended half-life (ranging from 14 to 24 hours) permits once-daily dosing, which can improve adherence in pediatric settings. More significantly, nadolol does not cross the blood-brain barrier due to its hydrophilic nature, potentially reducing neuropsychiatric side effects such as sleep disturbance and irritability, which are symptoms commonly reported with propranolol [27]. Furthermore, because nadolol is renally excreted and minimally metabolized by the liver, it may be advantageous for infants with hepatic immaturity, although caution is warranted in cases of renal impairment [5,25].

Available safety data on nadolol, while more limited than for propranolol, suggest a favorable tolerability profile. Bernabeu-Wittel et al. reported improved sleep quality and behavioral symptoms in children switched from propranolol to nadolol, with no major adverse events documented [27]. Nevertheless, isolated case reports have underscored the need for vigilance. McGillis et al. reported a fatal overdose in a 10-week-old infant, emphasizing that while nadolol may be safer pharmacologically, it still requires careful titration and monitoring [28].

Case-Based Observations and Subpopulation Use

Real-world clinical use supports nadolol’s application in settings where propranolol is contraindicated or poorly tolerated. Yang et al. retrospectively evaluated infants with subglottic hemangiomas and found that nadolol was effective with fewer cardiovascular side effects [24]. Though their sample size was small, the study contributes to a growing number of reports suggesting that nadolol is a feasible alternative for airway-involved or function-threatening IHs. These findings highlight nadolol’s potential role as second-line therapy or even first-line therapy in high-risk populations.

Risk-Benefit Considerations

While the breadth of clinical evidence on nadolol remains narrower than that for propranolol, its pharmacologic advantages and early efficacy data offer a compelling rationale for its use in selected patients. The long half-life, low CNS penetration, and favorable behavioral tolerability position nadolol as a strong candidate in cases where propranolol is contraindicated, poorly tolerated, or poses increased risk. However, due to nadolol’s renal excretion, careful patient selection and dosing adjustments remain crucial, particularly in neonates or infants with renal immaturity. Additionally, long-term data on neurodevelopmental safety, optimal dosing schedules, and use across diverse IH subtypes remain insufficient, underscoring the need for further prospective studies.

Future directions

Future research on nadolol for IH should focus on establishing its long-term safety and efficacy in pediatric populations. While preliminary studies suggest comparable therapeutic outcomes to propranolol with potentially fewer CNS-related adverse effects, large-scale, multicenter clinical trials are necessary to confirm these findings [4,27]. Additionally, further investigations are needed to assess nadolol’s safety profile, particularly in infants with comorbid conditions or those requiring long-term treatment, to ensure it remains a viable alternative. Dose and treatment regimen optimization is also an area to be explored. Currently, standardized guidelines for nadolol use in IH are lacking, leading to variability in clinical practice. Future studies should aim to determine the most effective dosing strategies, balancing therapeutic benefits while minimizing adverse effects. Research on treatment duration, tapering off protocols, and other combination therapies may also further enhance nadolol’s role in IH management.

Lastly, understanding long-term outcomes and potential broader applications of nadolol in IH treatment is essential. Longitudinal studies are needed to evaluate the risk of relapse and long-term complications, such as the potential impact on developmental milestones and overall cardiovascular health, in children receiving nadolol. Additionally, exploring its use in different IH subtypes may also expand its therapeutic application. By addressing these gaps, future studies can solidify nadolol’s role as a safe and effective alternative for infants who cannot tolerate propranolol.

## Conclusions

Nadolol offers a compelling alternative for the treatment of IHs, particularly in patients for whom propranolol is unsuitable. Available evidence supports its comparable efficacy to propranolol, with a pharmacologic profile that may confer meaningful safety advantages, including reduced CNS penetration and once-daily dosing. These attributes have the potential to improve both tolerability and treatment adherence in pediatric patients. While current findings are promising, defining nadolol’s optimal role will require well-designed, adequately powered clinical trials that assess long-term outcomes and establish standardized treatment protocols.
